# Welcome to *BJC Reports*

**DOI:** 10.1038/s44276-023-00001-1

**Published:** 2023-06-22

**Authors:** Marc Tischkowitz

**Affiliations:** Department of Medical Genetics, University of Cambridge, Cambridge, UK

It is my great pleasure to be part of the launch of a new sister journal to the *British Journal of Cancer*, *BJC Reports*. The *British Journal of Cancer* (*BJC*) is an esteemed journal that has been publishing in the field of cancer research for more than seven decades. During that time, we have seen enormous advances, yet there is still much to do. The idea of a sister journal to the *BJC* originated from the observation that many quality submissions to the *BJC* end up being rejected due to its threshold. Our aim with *BJC Reports* is to provide a fast and efficient avenue for publication of such manuscripts so that they can also benefit from the reputation and superb resource behind the *BJC*.

Most researchers will be aware of the explosion in journals over recent years and you may ask is there really need for another cancer journal? Much has been achieved since Richard Nixon announced the war on cancer over half a century ago. Despite this progress, the overall burden of cancer increases as the population gets older as more cancers are diagnosed in lower income countries due to changes in demographics and lifestyle habits. There is much work still to do in these areas and in those cancers, such as pancreas, where the survival rates remain disappointingly low.

Over that time there has been more than a twelvefold increase in the annual number of cancer-related published articles with a trend away from focusing on positive results in subscription-only journals to wider and faster dissemination of all relevant and robust findings (both positive and negative) in fully open access journals. Our aim is for *BJC Reports* to be part of shaping that trend over the next half century of cancer research which promises to be even more exciting than the previous 50 years, driven by huge advances in data science, artificial intelligence/machine learning, genomics and immunotherapies. Through the close relationship with the *BJC*, there is excellent potential to develop a strong sister journal and thereby enhance the potential for publication of high-quality research.

With *BJC Reports* we aim to provide a fast and efficient review process allowing publications, if accepted, to be rapidly published*. BJC Reports* is a 100% open access journal which is increasingly important in these days of data democracy.

Anyone familiar with the world of scientific publication will be aware of the unchecked growth predatory journals over the last decade that have a malign influence over the field. Those irritating emails from these journals that don’t quite make it to your junk folder can be easily dismissed by experienced researchers but can be confusing to those starting out in the field—a few times I’ve had students asking me for advice about this when their inbox gets bombarded following their first publication. Some of these journals have websites that look plausible, but on careful inspection it becomes evident that they are fronted by a rogue publisher. How can the author or reader tell what is real and what is fake, especially if the journal is new? Most of the time this will become apparent when looking at the editorial board and publishing house behind the journal. At *BJC Reports* we are fortunate to be supported by the huge resources of Springer Nature, one of the largest and best scientific publishers, whose extensive experience will underpin the success of this new journal.

As well as publishing important new research findings, we will foster a strong educational component to the journal. We will encourage not just general reviews but also mini-reviews focusing on a particular question or controversy in cancer. This will be an opportunity for leaders and newcomers to the field to challenge established dogma or to put forward new hypotheses which will generate debate and discussion. Another angle we would like to promote is the idea of case-based reviews, where a specific controversy in management or treatment is used as an example to drill down to the data behind that question. All too often assumptions are made in management of cancers because they have always been that way but when one looks at the data behind those assumptions it is often based on old studies that may have been flawed. An example in my own field was the long-standing idea that risk reducing salpingo-oophorectomy in *BRCA1* and *BRCA2* mutation carriers also led to a reduction in the risk of breast cancer. This dogma was established following the publication of convincing studies in the mid-2000s but these were questioned 10 years later when new, contrary findings led researchers to revisit the statistical methodology used at the time.

So please reach out to me with your exciting manuscripts, perspectives, ideas for reviews, collections, and suggestions for areas that you would like *BJC Reports* to focus on. Together we can grow *BJC Reports* into a cutting-edge journal that befits the depth and breadth of the astonishing world of cancer research we are all privileged to be part of.

